# Unveiling the Core Competencies in Neuroscience Nursing Within the Context of Italy: A Qualitative Phenomenological Analysis†

**DOI:** 10.3390/healthcare12202068

**Published:** 2024-10-17

**Authors:** Antonio Bonacaro, Elisa La Malfa, Michele Minari, Rachele La Sala, Giovanna Artioli, Federico Cortese

**Affiliations:** 1 Department of Medicine and Surgery, University of Parma, Via Gramsci 14, 43126 Parma, Italy; elisa.lamalfa@unipr.it (E.L.M.); giovanna.artioli@unipr.it (G.A.); 2 Azienda Ospedaliero Universitaria Ospedale Maggiore, Via Gramsci 14, 43126 Parma, Italy; mminari@ao.pr.it (M.M.); rlasala@ao.pr.it (R.L.S.); federico.cortese@unipr.it (F.C.)

**Keywords:** neuroscience nursing, competencies, personalized care, neurological disorders, substance addiction

## Abstract

**Background**: In 2008, the European Association of Neuroscience Nursing (EANN) initiated the NeuroBlend™ project, which aimed to delineate the roles and competencies of neuroscience nurses across Europe. This project resulted in the development of the European Competence Profile (ECP), intended to standardize educational pathways and professional recognition for this specialized field. **Aim**: This study examines the relevance of neuroscience nursing practices to the competencies outlined in NeuroBlend™ across different Italian care settings, including multiple sclerosis, pediatric neurosurgery, pediatric neurology, Alzheimer’s disease, and substance addiction. **Methods**: A phenomenological approach was employed, utilizing focus groups to gather the perspectives of nurses on the competencies defined by the NeuroBlend™ model. The focus groups were conducted from May 2022 to September 2022. The data collected from the focus groups were analyzed using thematic analysis. **Results**: Thirty nurses participated in the focus groups. Thematic analysis revealed the core competencies, such as communication, empathy, flexibility, and reflective skills, as critical in all care settings. The main themes that emerged were relationships with patients and families, personalized care, and continuity of care. **Conclusions**: The competencies outlined in the NeuroBlend™ framework are vital to the role of neuroscience nurses in the settings studied and remain highly relevant in the context of Italian healthcare.

## 1. Introduction

Neuroscience nursing is a specialized field that demands advanced expertise, critical thinking, and continuous education to deliver optimal care for patients with neurological disorders. The increasing complexity of conditions, such as brain tumors, traumatic brain injuries, strokes, and neurodegenerative diseases like Parkinson’s requires neuroscience nurses to consistently enhance their skills through rigorous training and professional development. These disorders pose not only clinical challenges but also require holistic, patient-centered care that addresses the psychological and emotional needs of patients and their families [1].

Recent studies highlight the importance of career development strategies, including high-quality education and specialized certifications, in improving nursing practices. For both novice and experienced nurses, these strategies are crucial to staying current with the latest advancements in neurological care, ultimately enhancing patient outcomes [2]. Neuroscience nurses play a vital role in critical care units, stroke centers, and neuro-intensive care settings, where rapid decision-making and life-saving interventions are crucial. The fast-paced innovations in neurological treatments further emphasize the need for continuous learning, collaborative care, and leadership development in this nursing specialty [3].

Nurses in this field often confront various challenges, from the emotional strain of managing life-threatening conditions to the risk of burnout due to the demanding nature of their work. Conditions like strokes and brain tumors require timely interventions, adding to the emotional pressure. Moreover, nurses frequently encounter verbal aggression from patients, particularly in cases involving neuropsychiatric complications or substance abuse. These challenges underscore the need for peer support systems and training programs that focus on stress resilience, coping strategies, and de-escalation techniques [3]. By building resilience and fostering a supportive work environment, neuroscience nurses can mitigate the negative impact of these stressors on their mental health.

A critical component of neuroscience nursing is interprofessional teamwork, which has been shown to improve healthcare outcomes. Collaboration with neurologists, physical therapists, and social workers allows for a more comprehensive approach to patient care. Advanced practice nurses often work closely with these professionals to maintain high standards of care [4]. Research shows that teamwork not only enhances nurses’ confidence in handling complex cases but also highlights the role of educational programs in boosting their proficiency [5]. The ability to function as part of an interdisciplinary team is a core competency in neuroscience nursing, enabling the integration of diverse perspectives in patient management.

Certifications and specialized training are essential for maintaining proficiency in neuroscience nursing. Programs focused on neuro-resuscitation, stroke care, and pediatric neurology allow nurses to develop and demonstrate advanced skills. These certifications not only improve clinical competency but also validate expertise in the field [4]. Organizations like the American Association of Neuroscience Nurses (AANN) have developed certifications, such as the Certified Neuroscience Registered Nurse (CNRN), which help standardize the level of care across various healthcare settings [6].

Mentorship and interdisciplinary collaboration play a pivotal role in the professional development of neuroscience nurses. These initiatives foster critical thinking, communication, and leadership skills. For example, mentorship allows for the transfer of knowledge and best practices from experienced nurses to newer ones, while interdisciplinary collaboration strengthens nurses’ ability to provide evidence-based, patient-centered care. These efforts contribute to reduced stress, higher job satisfaction, and improved patient outcomes [7].

In addition, neuroscience nurses’ roles extend beyond clinical care to include rehabilitation and patient transitions, where they are key in educating patients and caregivers. Nurses often ensure that patients and families are well-informed about managing health conditions post-discharge. In this context, health literacy and effective communication are vital for promoting successful rehabilitation and recovery [8]. Family involvement in patient care can vary widely depending on cultural, social, and economic factors. In some regions, families play a crucial role in supporting patient recovery, adding further complexity to the nurse’s role [9].

Globally, neuroscience nursing continues to evolve with the advent of new technologies and treatments. Initiatives like the NeuroBlend™ project, which aims to standardize neuroscience nursing roles and competencies across Europe, highlight the importance of cohesive education and practice approaches. Such initiatives help ensure that nurses are well-prepared to manage the rapidly evolving landscape of neurological care [10]. Moreover, the European Competence Profile (ECP) serves as the guiding framework for evaluating these competencies.

Despite the growing recognition of the importance of specialized competencies in neuroscience nursing, there remains a significant gap in the literature regarding how these competencies are applied in practice across different care settings. Most existing studies focus on the general competencies of nurses in specialized fields, but there is a lack of comprehensive research examining the specific skills required in neuroscience nursing, particularly in the context of varied neurological conditions, such as multiple sclerosis, pediatric neurosurgery, Alzheimer’s disease, and substance addiction. Additionally, while the NeuroBlend™ model has been established to standardize neuroscience nursing roles and competencies across Europe, there is limited empirical evidence assessing its relevance and implementation in diverse clinical environments, especially within the Italian healthcare system.

This study aims to map the competencies of neuroscience nursing within the Italian healthcare context, particularly in managing multiple sclerosis (MS), pediatric neurosurgery, pediatric neurology, Alzheimer’s disease, and drug addiction. Furthermore, this work seeks to assess how well the competencies outlined by the NeuroBlend™ project align with the challenges faced by Italian neuroscience nurses.

## 2. Materials and Methods

A phenomenological approach was adopted, utilizing focus groups to capture nurses’ perspectives on the competencies outlined by the NeuroBlend™ model. Focus groups were selected as the sole data collection method for this study due to their unique ability to generate in-depth insights into the participants’ shared experiences, attitudes, and perceptions within a specific context. In qualitative research, particularly in phenomenological studies, the goal is to explore the lived experiences of individuals, and focus groups provide an interactive setting that fosters dynamic discussions, allowing participants to reflect on and build upon each other’s responses.

The sampling strategy for this study was designed to ensure the inclusion of a diverse and representative group of neuroscience nurses, with the aim of capturing a broad spectrum of experiences related to the NeuroBlend™ competencies. A purposive sampling approach was adopted, targeting nurses who work in specialized neurological care settings, including multiple sclerosis, pediatric neurosurgery, pediatric neurology, Alzheimer’s disease, and substance addiction [11]. This was carried out to ensure that the study includes perspectives from a variety of neurological subfields, each of which may present unique challenges and competency requirements.

The selection of participants was based on their level of experience in neuroscience nursing, categorized as:

➢ Junior nurses (2–3 years of experience)
➢ Skilled nurses (4–5 years of experience)
➢ Expert nurses (more than 5 years of experience)

Each focus group consisted of 6 nurses, 2 from each above-mentioned category; 5 focus groups took place totaling 30 participants. The contact details of the nurses were obtained from a mailing list provided by an Italian scientific society specializing in neuroscience nursing. Participants were voluntarily recruited by emailing the participant information sheet and the related consent form. The final sample of 30 participants, distributed across five focus groups, was deemed sufficient to reach data saturation, a point where no new themes or insights emerge from additional data collection. This size allowed for meaningful discussion while ensuring that all participants could contribute to the conversation.

Each focus group [12] was initiated by introducing topics, such as communication skills, education skills, flexibility, analytical skills, ethical considerations, initiative, empathy, stress resilience, and reflective skills. The focus groups were conducted from May 2022 to September 2022 via Microsoft Teams version—1449/1.0.96.2022051102. Subsequently, conversations were transcribed and anonymized to ensure confidentiality.

The data from the focus groups were analyzed using thematic analysis, following the systematic six-phase framework developed by Braun and Clarke [13,14]. This approach allowed for the identification of recurring themes that represented the competencies described by neuroscience nurses in the context of their work. The analysis process involved the following key steps:

1. Familiarization with the Data: Initially, all focus group discussions were transcribed verbatim and read multiple times by the research team. This stage was critical for gaining an in-depth understanding of the content and identifying initial impressions of recurring concepts and issues. This process ensured that the researchers were fully immersed in the data before beginning formal coding.
2. Generating Initial Codes: After familiarization, the researchers systematically worked through the transcripts, assigning codes to significant data segments that related to the study’s objectives. Coding involved identifying meaningful units of information, such as statements about competencies like communication, flexibility, or empathy. The coding process was carried out both manually and with the assistance of NVivo^®^ software version 1.7, a widely used qualitative data analysis tool, to ensure that the data were organized and coded consistently. This software facilitated the systematic comparison of coded segments across the transcripts and allowed for the efficient management of large volumes of qualitative data.
3. Searching for Themes: Once coding was completed, the researchers reviewed the coded data to identify broader themes that captured essential patterns related to the competencies discussed. This step involved grouping similar codes together into potential themes, such as communication skills, patient education, or flexibility in care delivery. The themes were chosen based on their relevance to the research questions and the frequency with which they appeared across different focus groups.
4. Reviewing Themes: After the initial themes were identified, the researchers reviewed them in relation to the coded data and the overall dataset. This stage involved ensuring that the themes were coherent, distinct from one another, and reflective of the data. Any themes that overlapped or were insufficiently supported by the data were refined or discarded. The team cross-checked the themes to ensure they accurately represented the participants’ perspectives and the phenomena under study.
5. Defining and Naming Themes: In this phase, the final themes were clearly defined and named to reflect the essence of each competency discussed by the participants. Detailed descriptions of each theme were developed, ensuring that they captured both the explicit content of the focus group discussions, and the underlying meanings conveyed by the participants.
6. Producing the Report: Finally, the thematic analysis was written up, providing a narrative of each theme supported by direct quotes from the participants. These quotes were used to illustrate the identified themes and to give voice to the nurses’ experiences in a nuanced manner.

To ensure the reliability and validity of the findings, several measures were taken, such as:

– Triangulation of Researchers: Multiple researchers were involved in the coding and analysis process. This triangulation ensured that different perspectives were considered during theme identification, reducing individual bias and enhancing the rigor of the analysis.
– Use of NVivo^®^ Software: The use of NVivo software allowed for a systematic and transparent coding process, enhancing the reliability of the analysis. The software helped in organizing the data, tracking the coding process, and ensuring that all relevant data were systematically examined.
– Inter-coder Reliability: The research team conducted regular meetings to compare codes and resolve any discrepancies. This process of consensus-building helped improve inter-coder reliability and ensured that the coding was consistent across the dataset.
– Peer Debriefing: To further enhance the validity of the findings, peer debriefing was conducted with colleagues who were not directly involved in the analysis process. This involved presenting the themes to external researchers to ensure that the interpretations were credible and grounded in the data.
– Member Checking: While member checking was not possible for all participants due to time constraints, selected participants were asked to review the themes and findings to verify that the interpretations aligned with their experiences. This helped to ensure the credibility and trustworthiness of the analysis.

By employing these strategies, the study ensured that the themes identified were both valid and reliable, providing a robust understanding of the competencies required in neuroscience nursing across different clinical settings.

## 3. Results

After transcribing the discussions among the neuroscience nurses in the five focus groups, thematic analysis was performed. The authors identified the main themes that emerged concerning the competencies of neuroscience nurses in the Italian context, as inspired by the Neuroblend™ model [13,14].

Below are the results of the five focus groups broken down by the competencies reported by participants: communication skills, educational skills, flexibility, analytical capability, ethical considerations, initiative, empathic capacity, stress persistence and reflection skills.

### 3.1. Communication Skills

Effective communication is recognized as a fundamental competency for nurses caring for patients with MS. It facilitates the establishment of a therapeutic relationship with both the patient and their family, allowing for the identification of individual needs and the provision of personalized care. Communication is particularly vital during the diagnostic phase and the early stages of the disease but tends to diminish as the patient becomes more self-sufficient in later stages.

Nurses highlight the importance of tailoring communication to each patient’s specific characteristics, fostering trust, especially in the case of pediatric patients and their families. Non-verbal communication, such as gestures and eye contact, is also essential in enhancing the nurse–patient connection, helping to recognize and address the emotional needs of the patient.

In summary, both verbal and non-verbal communication are considered indispensable in delivering high-quality care and adapting to the unique needs of each patient and their family context (Table 1).

### 3.2. Educational Skills

Education is critical for nurses, particularly in the management of MS, where patients and their families must be well-informed to facilitate effective self-management. Nurses view education as essential at every stage of care, with particular emphasis on critical moments during the disease’s progression. However, addressing personal and emotional concerns can pose challenges for nurses.

In pediatric care, education extends to parents, whose active involvement is crucial for successful treatment outcomes. Nurses emphasize the importance of equipping parents with the skills necessary to care for their child, although obtaining full cooperation can sometimes be difficult. While caregiver education is considered an invaluable resource, it is often complex to implement and must be tailored to the specific needs of both the patient and the unique context (Table 2).

### 3.3. Flexibility

Flexibility is another crucial competency for nurses, especially in the management of MS, where the unpredictable nature of the disease necessitates continual adaptation to patients’ evolving needs. Although flexibility is key to delivering personalized care and responding to unexpected situations, it is not always recognized or valued as such and may be influenced by the individual traits of each nurse. Additionally, applying flexibility can be challenging in highly structured environments, where standardization dominates care protocols. Flexibility heavily relies on effective teamwork and collaboration to ensure that care remains both integrated and tailored to the patient’s needs (Table 3).

### 3.4. Analytical Capability

During the focus group discussions, nurses emphasized the critical role of analytical skills in identifying patients’ needs, including those that may not be explicitly communicated, to provide truly personalized care. These skills, developed through experience and supported by specific tools, are essential at every stage of the care process, especially in delicate or sensitive situations. Nurses associate analytical ability with professional experience, highlighting the importance of balancing theoretical knowledge, practical experience, and the flexibility to apply these skills effectively. They further stressed that analytical skills are indispensable in tailoring care to each patient’s unique circumstances (Table 4).

### 3.5. Ethical Considerations

Patients have the right to make informed choices about their care, and nurses must respect these decisions by providing clear, necessary information with an empathetic approach that fosters open communication. Maintaining impartiality is crucial to avoid influencing the patient’s choices, though it can be challenging in emotionally charged situations.

The concept of the ‘professional duty’ of nurses was also discussed, with particular attention to the difficulty of maintaining emotional detachment while making ethically sound decisions. The need for greater social recognition of MS, similar to that seen with Alzheimer’s disease, was noted, along with a call for educational initiatives to enhance public understanding.

Lastly, respect for patients’ rights was emphasized, especially the need to balance honoring their wishes with the professional responsibility to ensure their well-being. This involves providing what is necessary for their health rather than simply acquiescing to their demands (Table 5).

### 3.6. Initiative

The nurse’s initiative is crucial in emphasizing the importance of treatment to patients and their families, ensuring active involvement of caregivers and relatives throughout the care process. The ability to recognize and manage various clinical situations, distinguishing between routine and acute conditions, is closely linked to the nurse’s analytical skills and contributes to improving patient care. A strong working relationship within the multidisciplinary team enhances the nurse’s capacity to take initiative, allowing for more autonomous decision-making. Key themes that emerged include the significance of ongoing training, communication skills, shared decision-making, personalized care, adaptability, and collaboration with caregivers. The nurse’s initiative often requires close coordination and alignment with the broader healthcare team to ensure seamless, patient-centered care (Table 6).

### 3.7. Empathic Capacity

Empathy is a fundamental competency for nurses, vital in fostering a trusting relationship with patients. However, the capacity for empathy can vary based on the individual characteristics of the nurse and is often influenced by personal experiences. Empathy becomes particularly crucial during pivotal moments, such as diagnosis, relapse or changes in treatment. Nurses must also manage their own emotions in emotionally charged situations to maintain professionalism. Although empathy can be cultivated and enhanced over time through training and support, it is essential for nurses to find daily motivation and encouragement to maintain an empathic approach. When patients feel understood, they are generally more open and engaged in their care, leading to better outcomes (Table 7).

### 3.8. Stress Persistence

Stress is a factor that can impact both individual nurses and healthcare teams, with potentially detrimental effects on the quality of care provided. Participants noted that unmanaged stress can hinder the ability to prioritize tasks effectively and emphasized that the well-being of healthcare professionals is essential to prevent negative outcomes for patients. Nurses also highlighted that experience plays a critical role in managing stress, as seasoned professionals tend to navigate stressful situations more effectively. However, the pressure to achieve perfection can intensify stress levels, underscoring the need for realistic expectations and support systems (Table 8).

### 3.9. Reflection Skills

Reflective practice is crucial in the nursing profession, as it enables nurses to evaluate and enhance the quality of care, set priorities, and maintain self-control. However, some participants warned against excessive reflection, which can lead to negative consequences, such as overthinking or burnout. While self-criticism and self-analysis are essential for identifying and correcting mistakes, these practices are often underutilized in healthcare settings, despite their significance for professional growth. Acknowledging personal limitations and reflecting on one’s performance promote continuous professional development, allowing nurses to refine their skills and improve the quality of care they provide (Table 9).

## 4. Discussion

A recurring theme across many competencies was the nurse–patient relationship, considered central to communication, education, analytical ability, initiative, and empathy. Building this relationship requires effective communication and empathy, particularly during initial patient contact. Price et al. underscore the role of clear communication in establishing a strong nurse–patient connection [15,16]. Once this relationship is formed, the entire care process revolves around the patient, making it indispensable to the quality of care. Similarly, relationship with family members is crucial, especially in cases of debilitating conditions where caregivers play a pivotal role. Effective communication with caregivers is essential for providing adequate care, and empathy helps reduce caregiver stress and anxiety by fostering understanding and support [17].

The relationship between the competencies of neuroscience nurses and the overall quality of healthcare services is fundamental to delivering high standards of patient care. Nurses’ advanced skills, including communication, analytical abilities, empathy, and flexibility, directly contribute to more efficient and effective care. These competencies ensure that nurses can respond rapidly to the complexities of neurological disorders while enhancing the accuracy of care interventions, leading to better health outcomes [18,19].

Moreover, the development of these skills fosters a more patient-centered approach, which is closely tied to the quality of healthcare services. Personalized care—rooted in the ability of nurses to understand the unique needs of each patient—ensures that treatment plans are tailored to individual conditions, improving patient satisfaction and engagement in their care [17]. Research has shown that, when healthcare providers emphasize personalized care, patients experience better recovery rates and improved overall well-being [15].

Efficiency in healthcare is also enhanced through well-developed nursing competencies. Nurses with strong skill sets are better able to manage time-sensitive tasks, reducing delays and improving the coordination of care within interdisciplinary teams. By optimizing care delivery, these competencies contribute to both cost-effectiveness and the overall quality of healthcare services [20]. Additionally, greater personalization in healthcare not only adds value by improving patient outcomes but also by fostering stronger relationships between nurses, patients, and their families. This, in turn, leads to higher patient trust and adherence to treatment plans [19].

Personalized care emerged as a fundamental theme in all the settings analyzed, closely linked to competencies, such as communication, education, and analytical skills. Care is tailored to the specific needs of each patient, as seen in several studies [18,19,21,22]. Communication and education are intertwined, with a focus on delivering clear and accurate information to patients while addressing any misconceptions, as emphasized by participants. This highlights the importance of a multidisciplinary approach, where neurologists concentrate on treatment while nurses prioritize improving the patient’s quality of life [15].

The variability of interventions depends on both the patient and the nature of the disease, which often manifests in phases with varying symptoms. This complexity demands flexible care, a point stressed by participants who associated the unpredictable nature of neurological diseases with the need for stress resilience [15,23]. Providing optimal personalized care requires an interdisciplinary team, where professionals from various fields collaborate to meet the comprehensive needs of the patient [17].

An interdisciplinary approach not only benefits patients but also supports nurses, who gain from working with other healthcare professionals, consulting literature, and employing new technologies [5,22,24]. Team communication is vital, and studies, such as that by Saposnik et al., demonstrate that nurse–doctor collaboration significantly improves patient outcomes [25].

Participants agreed that communication, education, and analytical skills are essential throughout the entire care process, although certain moments, such as diagnosis, demand specific competencies, like empathy [17]. Analytical skills and flexibility are crucial, underscoring the interconnectedness of these competencies, as reflected in the framework outlined by Eklund et al. (2019) [20].

The role of the nurse in neuroscience is defined by these competencies. As central figures in patient care, neuroscience nurses must communicate effectively with patients, their families, and the healthcare team. This enables them to educate and empower patients for self-management while identifying and addressing unexpressed needs through strong analytical skills [26]. Empathy and flexibility are key to navigating the unpredictable nature of neurological conditions, particularly in situations where clear communication of therapeutic decisions is critical [27].

However, the demands of the profession—such as heavy workloads, staff shortages, and emotional strain—can lead to stress and loss of self-control. Participants acknowledged this as an inherent challenge but emphasized the importance of managing it without compromising patient care. They highlighted reflection as a tool for prioritizing tasks and managing workload effectively [28].

Reflection was identified as a core competency that encapsulates all others. It enables nurses to evaluate the effectiveness of their interventions, refine their practices, and learn from both personal experiences and those of others. Notably, nurses recognized the importance of setting boundaries to reflection to avoid becoming overwhelmed, suggesting a correlation between reflection and experience [22,27,29,30]. This balance ensures that nurses continue to develop professionally while maintaining their well-being in a demanding field.

However, this study presents several limitations that should be acknowledged. The scope of this research was limited to a specific set of neurological conditions, including multiple sclerosis, pediatric neurosurgery, pediatric neurology, Alzheimer’s disease, and substance addiction. Other areas within neuroscience nursing, such as adult neurosurgery or neurorehabilitation, were not explored, which could provide a more comprehensive view of the competencies required in different subfields. The focus groups were conducted over a limited time frame (May 2022 to September 2022), which may not capture the evolving nature of clinical practice and competency development in neuroscience nursing over a longer period. Additionally, reliance on virtual focus group meetings (via Microsoft Teams) may have impacted the depth and dynamics of discussions due to the virtual format’s limitations in fostering open, face-to-face communication. Lastly, while the NeuroBlend™ project provided a valuable framework for evaluating competencies, it may not fully capture all the relevant skills required in rapidly evolving healthcare environments, where new technologies and approaches to patient care are continually emerging. 

## 5. Conclusions

Modern neuroscience nursing demands advanced competencies in assessment, intellectual processing, planning, and intervention, covering all dimensions of nursing care. The rapid evolution of the field calls for evaluation tools that reflect the growing complexity of intellectual processes, critical thinking, and clinical decision-making [10]. This study marks the first attempt to map the competencies of neuroscience nurses in Italy, using the NeuroBlend™ reference model as a foundation.

However, the contexts within neuroscience nursing are diverse, and this research has only examined a limited subset. Expanding the analysis to include models from other countries, such as the American, Canadian, and Australian frameworks, would be valuable in identifying additional competencies that could be incorporated into the development of a comprehensive Italian reference model. To create an inclusive Italian model that addresses all aspects of neuroscience care, it is crucial to involve experts and specialized nurses in fields such as adult neurosurgery, Parkinson’s disease management, headache treatment, neurorehabilitation, and neuro-resuscitation.

## Figures and Tables

**Table 1:** Communication skills—Main themes.

Care Settings	Main Themes	Verbatim
Multiple sclerosis	Relationship with patient and family	“through communication, through the interview, through a little bit um maybe questions that are not direct, but that are indirect, you can perceive the state of mind, the problems” (B2)
Pediatric neurosurgery	The relationship with the family	“defining communication with a parent, with a caregiver, with a family member of a child in neurosurgery. First and foremost, I would say that communication must be real and truthful” (E4)
Pediatric neurology	Non-verbal communication	“without saying a word, the way one enters the room, even a glance is enough to be able to create or not a bond with that person and to make the patient not feel judged, but free to express his or her feelings” (B6)
Alzheimer’s	Personalized care	“It is an advantage if you already know the patient, my colleague mentioned the facilities earlier, those are the places where you can best establish communication directly with the patient at the highest level” (C5)
Drug addictions	Communication can be verbal or non-verbal	“You need to have advanced skills because listening cannot be learned by reading a psychology book; you have to experience it, you have to live it, you have to be appropriately supervised because listening implies two people who enter into a relationship, a verbal relationship but also a non-verbal one” (F4)

**Table 2:** Educational skills—Main themes.

Care Settings	Main Themes	Verbatim
Multiple sclerosis	Education takes place at all times	“... health education is the basis probably, in addition to communication and especially because our patients are, they self-manage then, they go home and do everything themselves...” (B13)
Paediatric neurosurgery	Participation in care	“...this is with regard to the child with the parents, i.e., how much they are willing to do and participate.” (B13)
Paediatric neurology	When to educate	“(...) every behaviour we act is in some way conveying an educational message (...)” (A12)
Alzheimer’s	Caregiver education	“Caregiver education as a great resource because in 2022 we have to remember that in the round table of doctors, nurses, physiotherapists, speech therapists also include the caregiver (...)” (D7)
Drug addictions	Personalised education	“It would be important to draw up goals based on the person’s educational needs and objectives, to also give timeframes in the goals, (...) it could also be customized and agreed upon with the person assisted” (F11)

**Table 3:** Flexibility—Main themes.

Care Settings	Main Themes	Verbatim
Multiple sclerosis	Patient needs	“... it is impossible not to be flexible with this type of patient, because it is an evolving, chronic, disabling disease” (D19)
Pediatric neurosurgery	Coping with various situations	“Flexibility is part of the job, depending on the patient’s clinic you go in one direction or another...” (C9)
Pediatric neurology	Personalized care	“(...) you have to be able to diversify them more and more, everything has a thousand nuances, a thousand facets too, which is the beautiful and fascinating part of our profile but also the exhausting part” (A18)
Alzheimer’s	Lack of flexibility	“flexibility is difficult in a ward like an RSA, a rehabilitation facility, in an acute ward there is no flexibility, everything is standardised. The end result is to solve the acute problem that brought the patient there” (E9)
Drug addictions	Flexibility as customization of the care plan	“So for us, flexibility is being able to adapt instantly to a situation that can degenerate, but not only that, it is also thinking, looking beyond” (A12)

**Table 4:** Analytical Capability—Main themes.

Care Settings	Main Themes	Verbatim
Multiple sclerosis	Personalized care	“The nurse’s skill lies in understanding what the patient is not telling you and thus also the patient’s unexpressed need...” (B13)
Pediatric neurosurgery	Professional experience	“...with children one also needs, in my opinion, a lot of experience, i.e., in the sense that one gets this analytical capacity by seeing perhaps more children” (B32)
Pediatric neurology	When to apply analytical skills	“...then don’t rely only on what we have been told or only on our first impression because we need all the analytical capacity, we can get all along the way...” (C23)
Alzheimer’s	Personalized care	“Analytical skill lies in joining, creating a link between what is safe for the patient and what is right for that moment (...)” (A11)
Drug addictions	Analytical capacity supported by knowledge and study	“And how to be analytical? First of all through study and training, and then all this must be supported by a holistic view and a certain flexibility” (A15)

**Table 5:** Ethical considerations—Main themes.

Care Settings	Main Themes	Verbatim
Multiple sclerosis	Role of the nurse	“... surely our duty also ethically is to give answers, knowing for example the pharmacological properties, or what the effect or undesirable effects are” (D30)
Paediatric neurosurgery	Impartiality	“...very important, (...) the nurse, however, must also be able to remain impartial” (B43)
Paediatric neurology	The professional duty of the nurse	“ (...) you are the one who has to do what you have to do, it is not easy to define where the line is between what is right and what is wrong” (A31)
Alzheimer’s	Social recognition of the disease	“(...) specialists who give indications from the clinical point of view explaining a bit what Alzheimer’s is and what happens” (C15)
Drug addictions	Patient rights	“Being ethical in my opinion means respecting the patient’s wishes, but we are not the ones who have to do what the patient wants, but we give him what he needs (...)” (A16)

**Table 6:** Initiative—Main themes.

Care Settings	Main Themes	Verbatim
Multiple sclerosis	Communicating with family members	“... and his family members should definitely be involved, family members, caregivers or whoever” (D35)
Paediatric neurosurgery	Recognising and dealing with situations	“(...) you have to have a lot of initiative and above all you have to be able, in that case, both to calm the child down and to try to calm the relatives, and this is always done maybe through initiatives, ideas, solutions, problem solving (...)” (B49)
Paediatric neurology	Relationship with the multidisciplinary team	“the nurse is well integrated into the team, can afford to take more initiative, and is then supported by the medical team” (B43)
Alzheimer’s	Nurse training	“sometimes we are the ones giving directions so we have to have a very good training that allows us to understand (...)” (C18)
Drug addictions	Shared initiative	“If, on the other hand, we talk about all the time and also in situations where, in short, there are no emergencies, the initiative in my opinion is very important. An initiative that, however, I would add is shared, in the sense that we have said from the outset that we work alone, yes, but as a team” (C19)

**Table 7:** Empathic capacity—Main themes.

Care Settings	Main Themes	Verbatim
Multiple sclerosis	Individuality of the nurse	“...but there is a difference between the nurse who can “read” the patient, they are not books, let’s be clear, but who can pick up on well-being, malaise, daily variations in mood (...)” (E32)
Paediatric neurosurgery	Understanding one’s own emotionality	“(...) with time may the management, the emotionality with many exercises, with much counselling, with many meetings with experts on this issue, be enhanced and improved (...)” (D28)
Paediatric neurology	The empathic relationship in care	“(...) every day, I always meet different children, different situations and every day is different from the one before and this gives me the strength to maintain that empathic relationship” (C33)
Alzheimer’s	Nurse’s experience	“let us remember that the job of ours and as in all jobs our staff can come in. We don’t just have a body but also everything else and if everything else is struggling we will also struggle at work” (C21)
Drug addictions	Empathy as a relationship tool	“Because the patient feels understood, he opens up more “ (B11)

**Table 8:** Stress persistence—Main themes.

Care Settings	Main Themes	Verbatim
Multiple sclerosis	Negative impact of stress on care	“our workload, besides being bureaucratic and physical, is also emotional...” (E36)
Paediatric neurosurgery	Managing work-related stress	“(...) we tend a little bit to accumulate what happened today, what happens tomorrow and then you get to a point where you can’t manage (...)” (C26)
Paediatric neurology	Team stress affects care	“All shifts are more or less stressful, but if you work in a closer team, you can cope with stress even better” (B58)
Alzheimer’s	Effects of stress	“as much as we always want to be able to do everything and do it precisely without failing because otherwise you feel like you’ve ruined everything, that too is stress. Only by accepting the fact that we are human can we come to terms with it” (A16)
Drug addictions	Experience helps	In my opinion there is a big, there is a very high level of stress that operators have to experience and therefore it is important to be aware of it in order to look for strategies to cope with it” (F32)

**Table 9:** Reflection skills—Main themes.

Care Settings	Main Themes	Verbatim
Multiple sclerosis	Reflection as the basis of the nursing profession	“...so reflecting on our competencies, support interventions, diagnoses, guidelines, helps us a lot to improve the quality of care.” (A43)
Paediatric neurosurgery	Self-criticism and self-analysis	“(...) it is also very important to realise what has been done, so that it does not happen again...” (D33)
Paediatric neurology	Knowing one’s limits	“...it is very challenging: sometimes it feels like we are in limbo, in a middle ground, where we are not speech therapists, but we are there when the patient will eat for the first time, we are not physiotherapists, but the patient will take the first steps with us, so it is heavy...” (C40)
Alzheimer’s	Reflecting on one’s own actions	“self-control is given to us by our knowledge, the more knowledge we have the more self-control we have” (D13)
Drug addictions	Professional growth	“Reflecting means havig a point of view about what I have done and what I have done wrong, and through these reflections I’m able understand myself and improve myself as well..” (A24)

## Data Availability

Dataset available on request from the authors.
